# Supplementary material for: Mining Thermophile Photosynthesis Genes: A Synthetic Operon Expressing Chloroflexota Species Reaction Center Genes in Rhodobacter sphaeroides
Source: Biomolecules. 2025 Oct 30;15(11):1529. doi: 10.3390/biom15111529 (PMC12650329; doi:10.3390/biom15111529)
Supplement: Supplementary file 1 [file biomolecules-15-01529-s001.zip › Figures S1-S4.pdf]

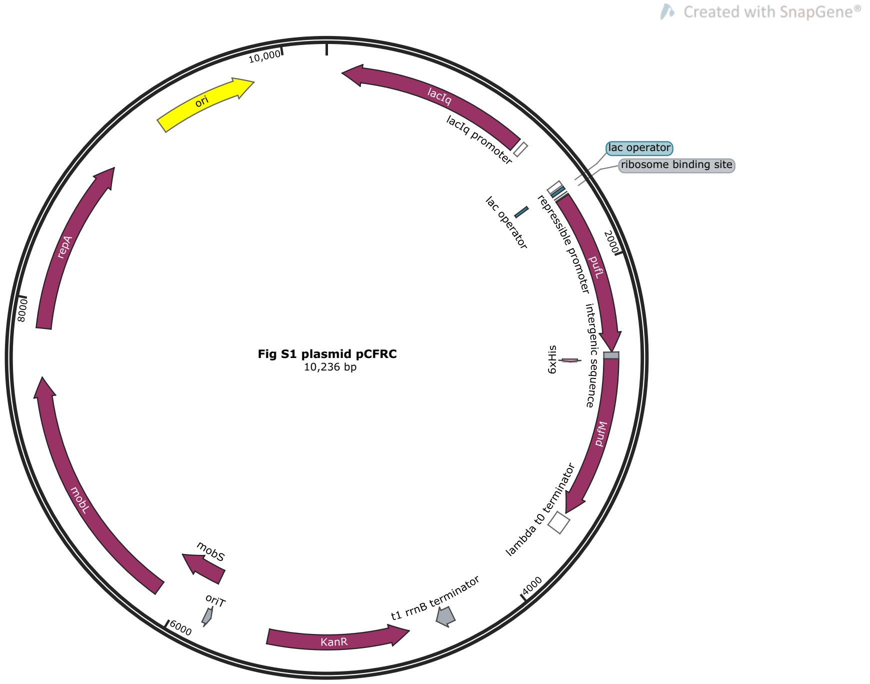

Figure S1. Genetic map of plasmid pCFRC.

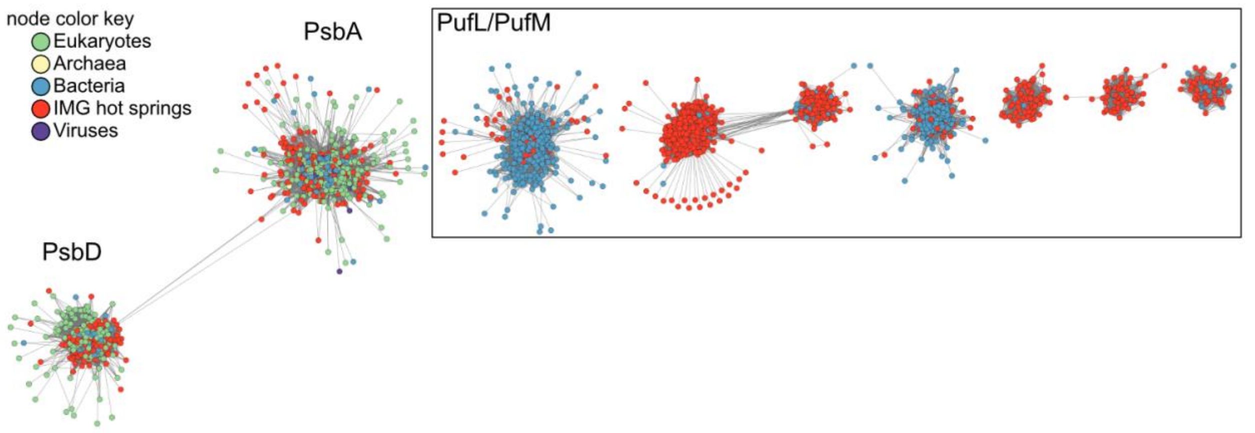

Figure S2. Identification of PufL and PufM proteins encoded by metagenomic samples from hot springs. Sequence similarity network of proteins with matches to PF00124. Nodes are colored according to the node key.

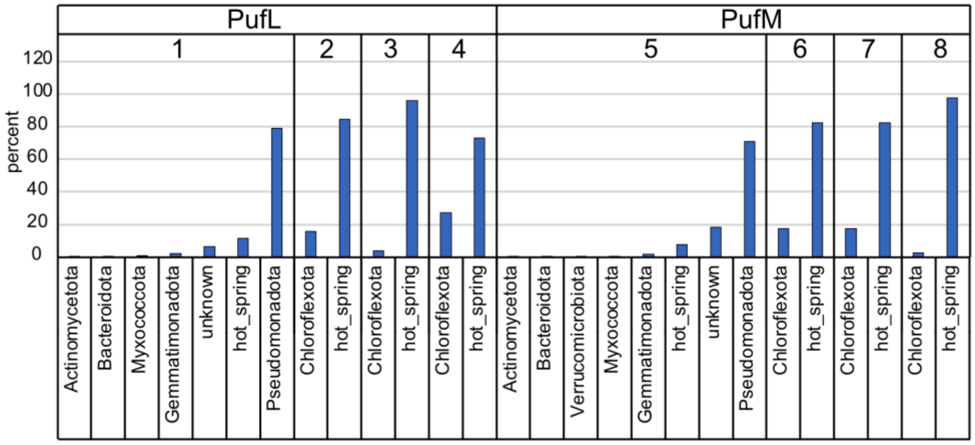

Figure S3: Representative taxa for each node in Figure 8. For each node in the PufL-PufM SSN, the number of known taxa were calculated from the node members. The most abundant known phylum was used as the representative taxon. The percentage abundance was calculated by dividing the number of nodes with a given representative taxon by the total number of nodes in a given cluster.

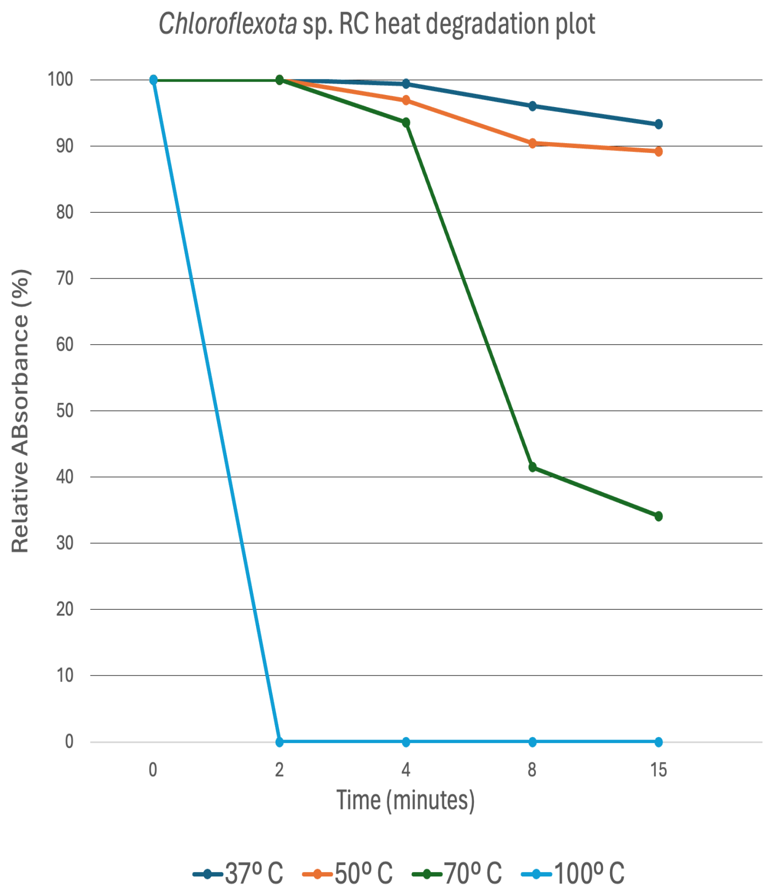

Figure S4. Change in absorbance of the RC accessory BChl peak at 810 nm as a function of time of incubation at the temperatures indicated.
